# Supplementary material for: Anderson localisation in steady states of microcavity polaritons
Source: Sci Rep. 2019 Dec 18;9:19396. doi: 10.1038/s41598-019-55673-y (PMC6920441; doi:10.1038/s41598-019-55673-y)
Supplement: Supplementary file 1 — Supplementary Information [file 41598_2019_55673_MOESM1_ESM.pdf]

Supplementary Information: Anderson localisation in steady states of microcavity polaritons

Thomas J. Sturges*[1], Mitchell D. Anderson[2], Adam Buraczewski[1], Morteza Navadeh-Toupchi[2], Albert F. Adiyatullin†[2], Fauzia Jabeen[2], Daniel Y. Oberli[2], Marcia T. Portella-Oberli[2], and Magdalena Stobińska[1]

[1]Institute of Theoretical Physics, University of Warsaw, ul. Pasteura 5, 02-093, Warsaw, Poland
[2]Institute of Physics, School of Basic Sciences, Ecole Polytechnique Fédérale de Lausanne, 1015 Lausanne, Switzerland

ABSTRACT

We present an experimental signature of the Anderson localisation of microcavity polaritons, and provide a systematic study of the dependence on disorder strength. We reveal a controllable degree of localisation, as characterised by the inverse-participation ratio, by tuning the positional disorder of arrays of interacting mesas. This constitutes the realisation of disorder-induced localisation in a driven-dissipative system. In addition to being an ideal candidate for investigating localisation in this regime, microcavity polaritons hold promise for low-power, ultra-small devices and their localisation could be used as a resource in quantum memory and quantum information processing.

1 Background renormalisation

To clarify the background renormalisation, in Fig. 1 we have provided the results before (top row) and after (bottom row) background renormalisation for (a) experiment, and (b) theory. In both figures the inset shows the background used for renormalisation. As explained in Methods, the background is obtained for the simulations by performing a separate simulation without mesas. For the experiment, the pump profile is obtained by fitting a Gaussian to a low-pass filtered copy of the image.

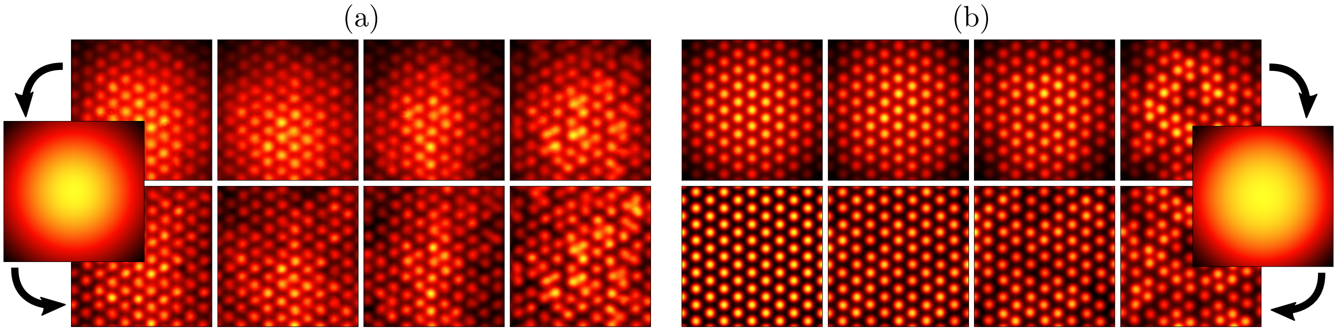

Supplementary Figure 1. Normalisation procedure. We show (a) experimental photoluminescence and (b) simulation results, exactly corresponding to those shown in Fig. 2 of the main text. The top row shows the results before background renormalisation, whereas the bottom row exactly corresponds to the figure in the main text. The insets show the background used.

*sturges.tom@gmail.com
†Current affiliation: Department of Physics, MIT-Harvard Center for Ultracold Atoms, and Research Laboratory of Electronics, Massachusetts Institute of Technology, Cambridge, Massachusetts 02139, USA

IPR as a function of disorder for several powers and detunings

We provide plots similar to Fig. 2(a) of the main text, for several powers. Crucially we recover the same monotonic increase in IPR with positional disorder.

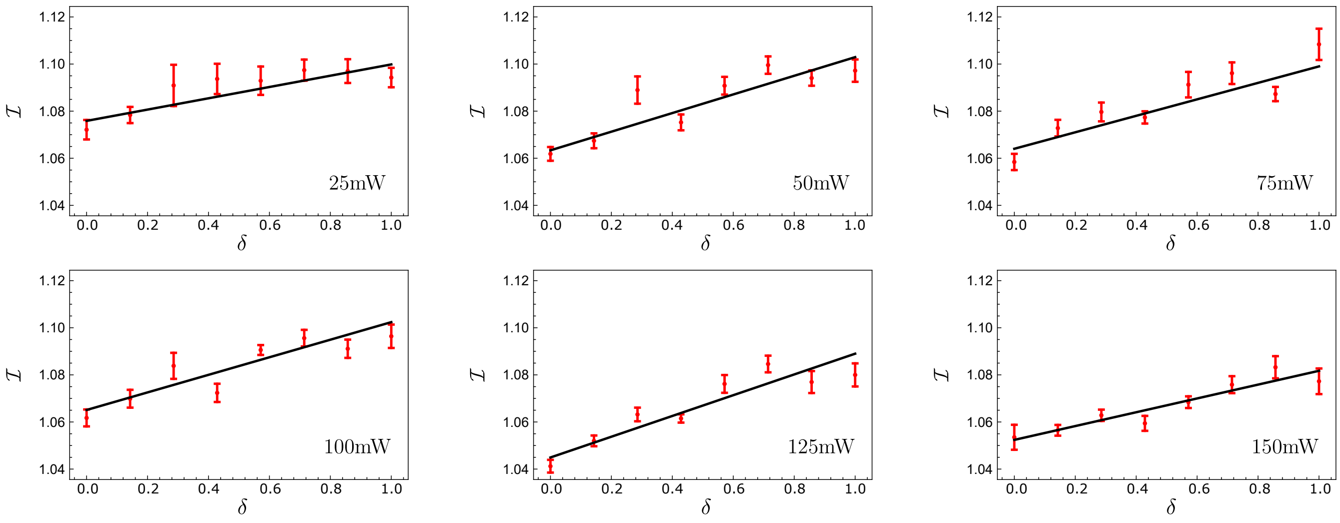

Supplementary Figure 2. IPR plots for a cavity-exciton detuning of 2meV. Error bars are the Standard Error of the Mean (SEM). A straight line is fitted with the least errors method, weighted by $1/(\mathrm{SEM})^2$

Variation of IPR with parameters of Gross-Pitaevskii model

In Supplementary Fig. 3 we provide a systematic study of the dependence of the IPR on the other parameters of the Generalized Gross-Pitaevskii model.

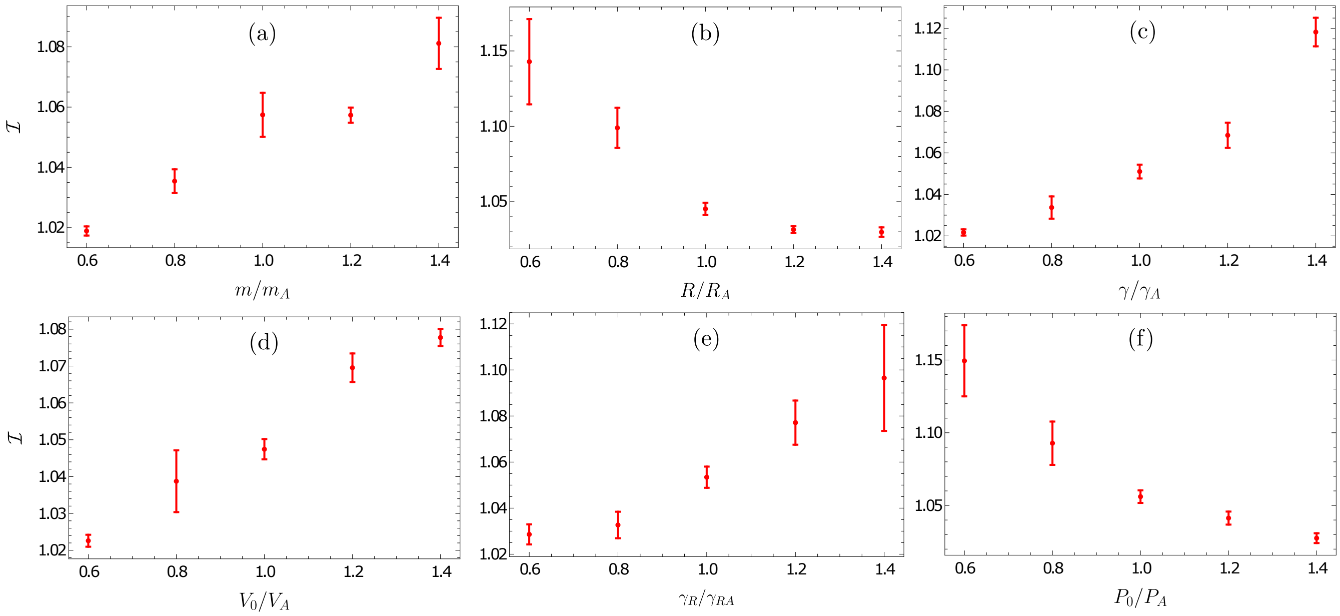

Supplementary Figure 3. Variation of IPR with parameters of Gross-Pitaevskii model. We examine the same model and parameters as used in Fig. 2(b) of the main text. However in panels (a-f) we tune each of the shown parameters from 60% to 140% of that value used in the main model. The varied parameters are given in units of those used in the main text, explicitly: $m_A = 5\times10^{-5}m_e$, $\hbar R_A = 0.4\mathrm{meV}.\mu\mathrm{m}^2$, $\hbar\gamma_A = 0.5\mathrm{meV}$, $\hbar g = 2.4\times10^{-3}\mathrm{meV}.\mu\mathrm{m}^2$, $V_A = 9\mathrm{meV}$, $\hbar\gamma_{RA} = 2\mathrm{meV}$, $P_A = 2\gamma_R\gamma/R$, $d = 0.25\mathrm{nm}$, and $L = 88\mu\mathrm{m}$.

Experimental spectra

In Supplementary Fig. 4(a) we display the photoluminescence spectra along the $k_x = 0$ axis, for the array without disorder. Crucially, we see that the polaritons behave as collective excitations that inherit their character from the lattice structure, rather than isolated mesa 'islands' with discrete flat energy bands. We extract a mesa-mesa interaction energy of $\hbar J = 0.11$meV by fitting the standard tight-binding dispersion $E(k) = -\hbar J f_{\mathbf{q}}$, where $f_{\mathbf{q}} = 2\cos(aq_y) + 4\cos(aq_y/2)\cos(\sqrt{3}aq_x/2)$ encodes the hexagonal geometry. For comparison we show the dispersion of a planar cavity in panel (b) with the same spacer thickness as the mesas.

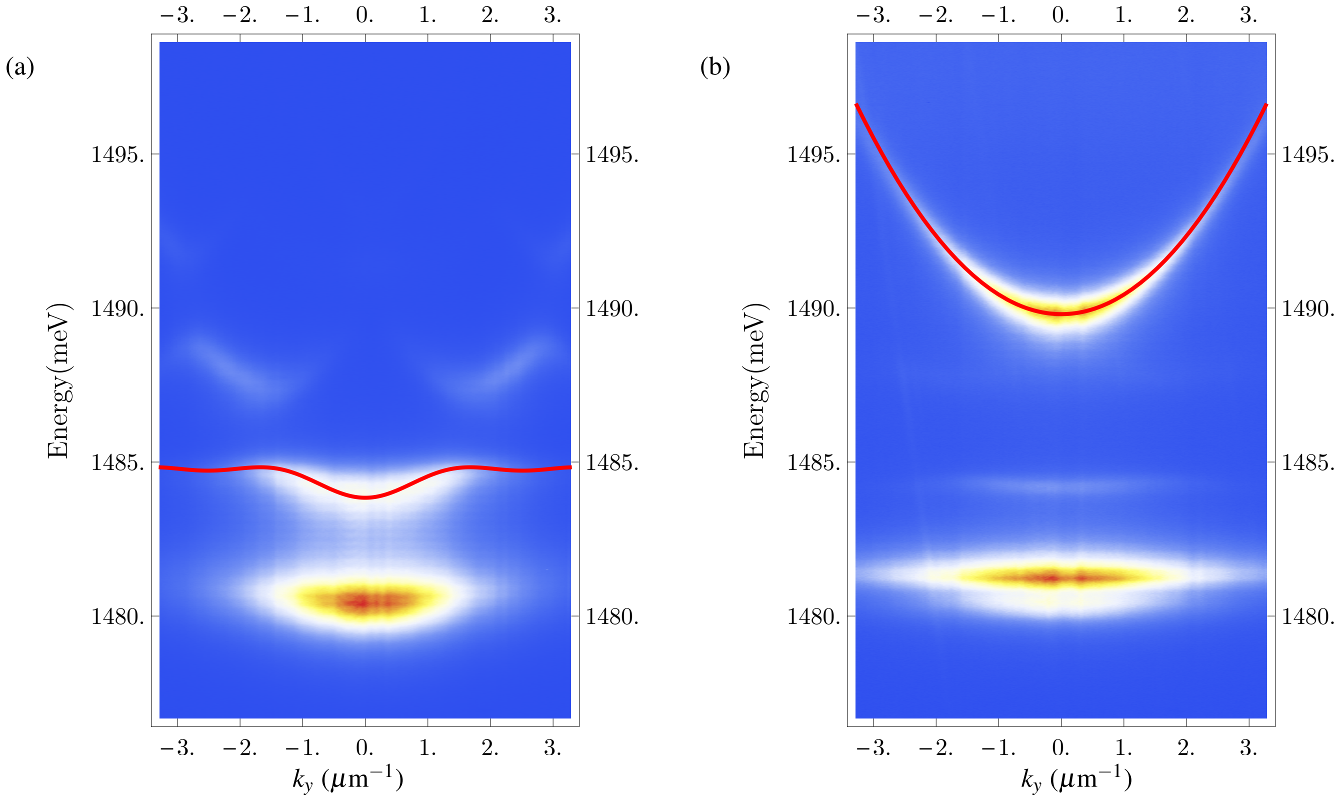

Supplementary Figure 4. Dispersion of microcavity. (a) Dispersion of a non-disordered hexagonal array of mesas at a detuning of 2meV. A tight-binding spectrum is fitted to the first upper-polariton branch (red curve), from which we extract a mesa-mesa coupling energy of $\hbar J = 0.11$meV. The reciprocal lattice vectors are $\mathbf{b_1} = (2\pi/\sqrt{3}a)(0,2)$ and $\mathbf{b_2} = (2\pi/\sqrt{3}a)(-\sqrt{3},1)$. (b) Dispersion of a planar cavity containing one mesa. A parabolic dispersion is fitted to the upper polariton. In addition we observe emission from two discrete states (upper and lower polariton ground state) coming from the single mesa.

Coupled mode model

To compliment the Generalized Gross-Pitaevskii model, we also investigated a model of coupled modes. In this case we assign a single nonlinear polariton mode to each mesa, and include nearest-neighbour interactions. The Hamiltonian of the system is

$$H = \hbar \sum_{n=1}^{N} \left[\omega_n a_n^\dagger a_n + g a_n^\dagger a_n^\dagger a_n a_n - \sum_{m=0}^{6} J_{mn} \left(a_n^\dagger a_{mn} + a_{mn}^\dagger a_n \right) \right] \quad (1)$$

where $a_n^\dagger$ is the bosonic creation operator for the polariton mode of the nth mesa, and $a_{mn}^\dagger$ that for its mth nearest-neighbour.

The polariton-polariton interaction strength is parametrised by g and the wavefunction overlap integral is modelled by

$$J_{mn} = J_0 \exp\left(\frac{1-s^2}{\tau}\right), \tag{2}$$

where a coupling energy of $J_0 = 0.11$meV is extracted from the experimental spectra and τ parametrises the decay of the wavefunction overlap integral as the separation of mesas is increased. Here,

$$s = \frac{|\mathbf{R}_m - \mathbf{R}_n| - 2r}{a - 2r}, \tag{3}$$

where r is the radius of the mesas. To simulate the dynamics of the system, we obtain the equations of motion from Supplementary Equation (1) and introduce a phenomenological damping rate γ_α, and gain rate $R\rho_n$ from the reservoir created by the nonresonant excitation

$$i\hbar\frac{d\alpha_n}{dt} = \hbar\left[g|\alpha_n|^2 + \frac{i\hbar}{2}\left(R\rho_n - \gamma_\alpha\right)\right]\alpha_n + \hbar\sum_{m=1}^{6} J_{mn}\alpha_m. \tag{4}$$

where α_n is the polariton wavefunction of the nth mesa. The equation of motion for the reservoir density is

$$\frac{d\rho_n}{dt} = P_0 - \rho_n\left(\gamma_\rho + R|\alpha_n|^2\right), \tag{5}$$

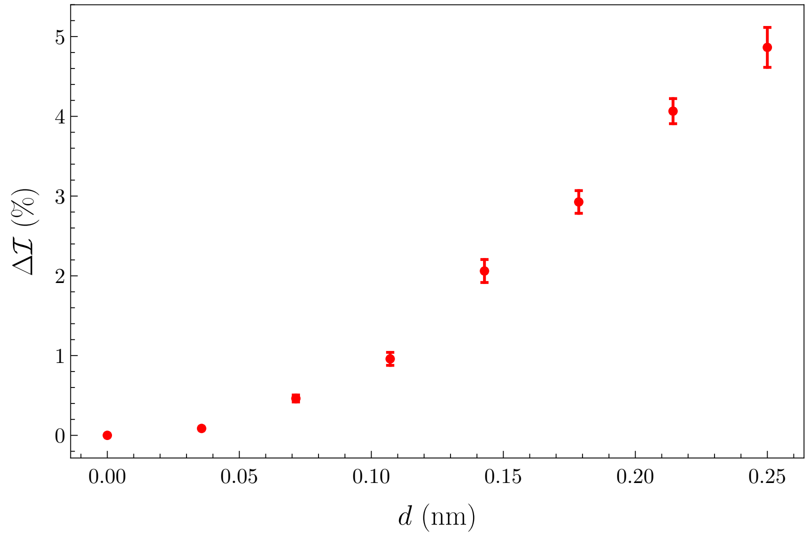

Supplementary Figure 5. IPR vs disorder for the coupled mode model. We show the percentage increase in the IPR as a function of the disorder d.

where γ_ρ is the decay rate of the reservoir and P_0 is the rate at which the reservoir is pumped by the laser. The equations were simulated for 1000ps which is much greater than the lifetime of a single polariton, so as to reach a steady state, and then the IPR was calculated as per equation 3 of the main text. The equations were solved numerically using the fourth-order Runga-Kutta method. Note that we assumed a homogeneous pumping here so we did not apply any data processing. In Supplementary Fig. 5 we observe a similar trend to that obtained for the Gross-Pitaevskii model in the main text.

The parameters of the simulation were $\hbar J_0 = 0.11$meV, $\tau = 6$, $\hbar\gamma_\alpha = 0.5$meV, $\hbar R = 2$meV.μm^2, $\hbar g = 0.015$meV.μm^2, $P_0 = 2\gamma_\alpha\gamma_\rho/R$, and $\hbar\gamma_\rho = 0.4$meV.
